# Information processing in the transcriptional regulatory network of yeast: Functional robustness

**DOI:** 10.1186/1752-0509-3-35

**Published:** 2009-03-19

**Authors:** Frank Emmert-Streib, Matthias Dehmer

**Affiliations:** 1 Computational Biology and Machine Learning, Center for Cancer Research and Cell Biology, School of Medicine, Dentistry and Biomedical Sciences, Queen's University Belfast, 97 Lisburn Road, Belfast, BT9 7BL, UK; 2 Center for Mathematics, Probability and Statistics, University of Coimbra, Apartado 3008, 3001-454 Coimbra, Portugal

## Abstract

**Background:**

Gene networks are considered to represent various aspects of molecular biological systems meaningfully because they naturally provide a systems perspective of molecular interactions. In this respect, the functional understanding of the transcriptional regulatory network is considered as key to elucidate the functional organization of an organism.

**Results:**

In this paper we study the functional robustness of the transcriptional regulatory network of *S. cerevisiae*. We model the information processing in the network as a first order Markov chain and study the influence of single gene perturbations on the global, asymptotic communication among genes. Modification in the communication is measured by an information theoretic measure allowing to predict genes that are 'fragile' with respect to single gene knockouts. Our results demonstrate that the predicted set of fragile genes contains a statistically significant enrichment of so called essential genes that are experimentally found to be necessary to ensure vital yeast. Further, a structural analysis of the transcriptional regulatory network reveals that there are significant differences between fragile genes, hub genes and genes with a high betweenness centrality value.

**Conclusion:**

Our study does not only demonstrate that a combination of graph theoretical, information theoretical and statistical methods leads to meaningful biological results but also that such methods allow to study information processing in gene networks instead of just their structural properties.

## Background

The advent of high-throughput technologies in molecular biology has initiated an avalanche of data that possess considerable challenges to quantitative sciences providing statistical analysis methods [1]. Due to the fundamental insight that biological processes should be studied holistically [2-4] instead of reductionistically, systems based approaches are of central importance in this respect [5]. For this reason, it is no surprise that network related studies experience an enormous interest starting with the investigation of small-world [6,7] and scale-free [8,9] networks in the mid 1990's followed by numerous studies devoted to the analysis of complex network topologies and their properties in general [8,10-14]. It is interesting to note that many apparently different networks have similar properties. Most prominent example is the degree distribution. For example, the World-Wide Web, the Internet or biological networks are found to be scale-free [8,10,11,15,16] with respect to their degree distribution. In molecular biology, metabolic, transcriptional regulatory, signaling and protein networks have been studied extensively during the last years [4,17-19] to shed light on the functional organization of these complex gene networks [20]. In this context, *functional robustness *is considered a key player for our understanding regarding the interplay of *network structure *and *network dynamics *leading to the emergence of life as omnipresent around us [9,21-23].

For general networks, one of the first studies that has thoroughly investigated *structural robustness *of systems that can be represented as networks is from ALBERT et al. [24]. ALBERT studied the error and attack tolerance of synthetic as well as real world networks and compared random and scale-free networks, e.g., the World-Wide Web or the Internet. By using purely graph theoretical measures – the diameter of the network and the size of the largest connected component – they found that scale-free networks are much more robust against random errors than random networks but more vulnerable against directed attacks. In the context of gene networks the interest shifts from the *structural robustness *of the networks to their *functional robustness *because the ultimate goal is of course to gain insights into the function of a living cell or an organism respectively. On a time scale of a living organism the question of *functional robustness *has been addressed by [25-28]. For example, in [27] the dynamics of Boolean networks [29,30] were studied serving as a simplified model for the signal processing taking place in gene networks. As major result [27] found that fluctuations occurring inevitably within the system, e.g., due to the inherent noise present on a molecular level [31,32], can be suppressed by a suitable design of the overall network topology [27]. On an evolutionary time scale the *functional robustness *of gene networks has been studied by [33-35] considering directly the role selective pressure might play during evolution leading to observable patterns of, e.g., protein structures, gene expression or network structures as present in current organisms. In this paper we tie up with previous studies aiming to analyze the *functional robustness *of networks on a time scale of living organisms. By pointing out the time scale we want to emphasize that we do not investigate the *evolutionary robustness *of an organism. Instead, the major objective of this paper is to investigate the functional robustness of the transcriptional regulatory network (TRN) of *S. cerevisiae *with respect to single gene perturbations. As quantitative measure of *functional robustness *we suggest to use an information theoretic measure [36], previously used to study synthetic networks, that does not focus directly on structural changes of the network topology due to the perturbations but on the alterations of information flow, modeled as Markov Chain [37], within the network as consequence of the structural modifications. The advantage of information theoretic measures [38-40] is that the concrete underlying dynamics does not need to be specified precisely, instead, a qualitative model is enough to gain principle insights into common working mechanisms with regard to more elaborate biological models. General entropy measures for quantifying structural information in networks have been developed in [41,42]. For our study, we use the transcriptional regulatory network of yeast [43,44] and apply our information theoretic measure to identify genes that are crucial for the functioning of the organism in the sense that disruptions of the transcriptional regulatory network are experienced strongest by these genes. For this reason we call these genes *fragile*. In this paper, we quantify our results by connecting these to the list of known so called *essential *genes of yeast [45] to demonstrate that our predictions are biologically meaningful.

## Methods

In this section we present the information theoretic measure we use to analyze the transcriptional regulatory network of yeast to study its functional robustness.

### Markov chains

We approximate the information flow in the network as a Markov chain. A Markov chain is a Markov process that is discrete in time and space. We define a Markov process by using a given network topology *G *and the plausible assumption that all possible interactions are equal likely. Plausible in this context does not necessarily mean that this corresponds best to the real situations, it means that it is the most simple and unbiased assumption one can make. For simplicity, we further assume the Markov process to be of first-order

(1) *T*(*X*_*t*+1 _= *j*|*X*_*t *_= *i*_*t*_, ..*X*_1 _= *i*_1_) = *T*(*X*_*t*+1 _= *j*|*X*_*t *_= *i*_*t*_).

That means, the transition probability *T *depends only on the last state and not on states that are further in the past.

**Definition 1: ***The transition probability T for a Markov chain of first-order for a network G with adjaceny matrix A is defined by*

(2)

*for all i, j *∈ *V*.

Here *k*_*i *_= ∑_*j *_*A*_*ji *_is the degree of node (gene) *i *in the network and *A*_*ij *_is a component of the adjacency matrix indicating if node *i *is connected with node *j *(*A*_*ij *_= 1) or unconnected (*A*_*ij *_= 0). *V *denotes a set comprising all genes.

### Single gene perturbations

In this paper we study the effect of single gene perturbations on the information processing in the transcriptional regulatory network of yeast. Formally, we define perturbations in the following way.

**Definition 2: (Single gene perturbations) ***If a gene k in network G is perturbed than all outgoing and incoming edges from this gene are deleted. In addition, one self-connection is introduced*.

**Figure 1:**
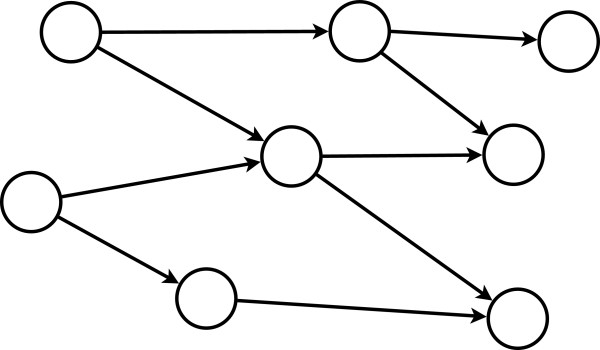
**A graph depicts the flow of information in the network**.

**Figure 2:**
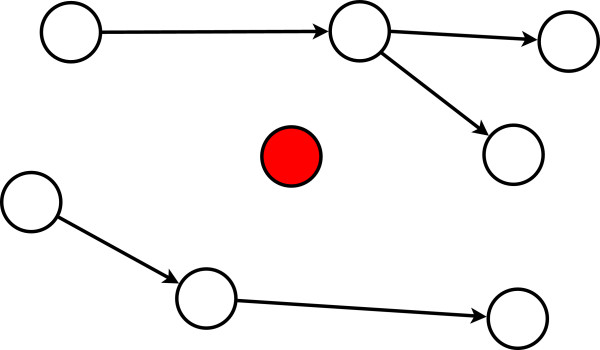
**Perturbing the node shown in red leads to a breakdown of communication between the red node and all other nodes**. However, there is still an information flow in the remaining network.

In Fig. 1 and 2 we visualize a single gene perturbation. One can see that the perturbed gene (shown in red) does no longer participate in the information processing in the network. However, the remainder of the network is still structurally intact and capable to transmit signals. Hence, a single knockout can be considered as a perturbation and the modified communication among the remaining genes can be studied principally provided there is a measure to quantify these alterations. This measure is given in the next subsection.

### Asymptotic Communication

The information theoretic measure we use to capture the asymptotic behavior of information processing evaluates the deviation of the unperturbed (or normal (n)) state from the perturbed (p) state caused by the perturbation of gene *k*. We use the the relative entropy also known as Kullback-Leibler (KL) divergence *D *[46,47] to quantify this deviation. Our asymptotic measure is given by the following definition.

Definition 3: (Asymptotic information change)

(3)

Here  and  are stationary distributions obtained by

(4)

(5)

The Markov chain given by *T*_*k *_corresponds to the process obtained by perturbing gene *k *in the network. The stationary distribution  of the perturbed (p) network obtained by perturbing gene *k *and starting from the initial distribution,

(6)

depends on *i *because we use the Kronecker delta, which is one for *i *= *m *and zero otherwise, as initial condition. The reason therefore is we consider *i *as starting point for the spread of information in the network. The interpretation for the unperturbed (normal (n)) distribution  is correspondingly. We want to note that due to the directedness of the network the Markov process is not ergodic which results in a dependence of the asymptotic distributions  and  on the initial distribution . For this reason it is important to use |*V*| - 1 (starting from *k *is excluded because the perturbed gene has no longer outgoing edges) different initial distributions  to evaluate *D*_*ik*_. That means Eq. 3 defines the components of a matrix and the interpretation of *D*_*ik *_is that the index *k *correspond to the deletion of gene *k *and index *i *referes to the initial distribution  = *δ*_*i, m*_. The diagonal elements *D*_*ik *_(*i *= *k*) are not defined.

## Results and discussion

### Data

For our analysis we use the transcriptional regulatory network of yeast [43,44] which is a directed, unweighted network. From this network we extract the weakly connected component consisting of 3357 genes and 7230 interactions. The weakly connected component of a network is defined as the subnetwork that connects every pair of nodes by at least one directed path. That means for every pair of genes the weakly connected component ensures that communication (at least in one direction) between these genes is in principle enabled. This is an important characteristic because in our analysis we are aiming to quantify modifications of the communication among genes due to perturbations. Hence, if there would be no path between genes such an analysis would not be sensible.

On a practical note, we want to remark that our theoretical analysis described in detail in the next section is computationally expensive because we perform single gene perturbations for all genes in the network. That means, we do not just analyze one network with our method but as many as genes in the network. Hence, the results presented in this article are obtained by analyzing 3357 networks. It is clear that this is getting more and more demanding computationally by increasing the number of genes in the network. From our simulations we found that networks with several thousand nodes can be studied within reasonable time whereas larger networks would require more algorithmic attention to reduce the computation time.

## Results

Now we study the asymptotic behavior of the transcriptional regulatory network of yeast regarding information propagation under the influence of single gene perturbation.

For the normal (unperturbed) and perturbed network topology of the transcriptional regulatory network we determine Markov chains from which we calculate the stationary distributions. The perturbations correspond to single gene perturbations and the Markov chains are obtained as described in the methods section. From the resulting stationary distributions of the Markov chains we calculate the Kullback-Leibler divergence *D*_*ik *_= *D *(||) for all genes *i *∈ *V *and perturbations *k *∈ *V *with *i *≠ *k*. We want to note that due to the directedness of the network the resulting Markov process is no longer ergodic. Hence, information sent from different genes can results in different stationary distributions. For this reason, we use all *N *genes consecutively as sender gene. This is reflected by the index *i *in Eq. 3 corresponding to the gene from which the information was sent initially. On a mathematical note we want to remark that the network does not need to be disconnected to result in a non-ergodic Markov chain. However, the need to consider different initial conditions to study the behavior of the resulting stationary distributions meaningfully remains also true in this case.

We begin our analysis by investigating if the asymptotic results summarized by *D*_*ik *_can be connected to local, structural properties of the genes in the network. For this reason we determine all genes with

(7)

and calculate the correlation with the in- and out-degree vector of the network. More precisely, we calculate Spearman's rank-order correlation coefficient [48] between the rank ordered vectors to decide if the order in these vectors is statistically preserved. For the in-degrees we obtain a correlation of *r *= -0.39 and *p *= 6 *× *10^-9^, for the out-degrees *r *= 0.33 and *p *= 1 *× *10^-6^. Using a significance level of *α *= 0.05 indicates that both rank correlations are statistically significant implying that, e.g., high out-degrees correspond to high values of *D*_*i*_. These results seem plausible considering the following situation: For a given gene that is connected to all other genes (outgoing edges) it is clear, that an arbitrary knockout of a single gene effects with probability one an outgoing edge of this gene. Hence, this knockout will have an influence on the information processing of this gene. The strength of this influence can not be easily predicted given just this information, however, we will have an influence with probability one. Instead, a gene having very few outgoing connections has a lower probability that a single knockout effects one of its outgoing edges (Pr = *k*_*out*_/*N*_*p *_with *N*_*p *_the number of genes that can be perturbed). However, it is possible that the knocked out gene destroys some communication paths (secondary- or even higher-order effect if measured as Dijkstra distance [49]) and, hence, can still have a strong impact on the information processing. It seems to be reasonable to assume that the further away the knockout gene is from the starting gene (in Dijkstra distance [49]) the less the impact will be. This is a strong indicator that information processing on a systems level depends crucially on the information processing in a local environment of the gene that sends the information. We want to remark that in our analysis the number *D*_*i*_, given in Eq. 7, is a global measure, whereas the degree vector is a local measure. This result is interesting because it demonstrates that the local properties of genes, given by their local connectedness, which can be roughly summarized by their degrees, are not averaged away with respect to the stationary distribution of the Markov process. That means the local *connectivity signature *is still detectable in the asymptotic behavior. We will come back to this point in the discussions section because this is a non-trivial point.

**Figure 3:**
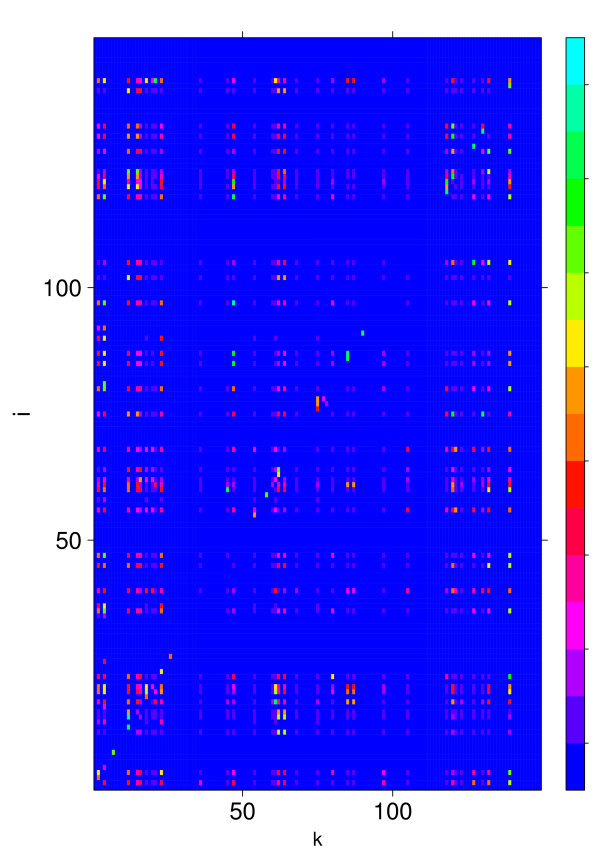
**Asymptotic information change *D*_*ik *_for all genes with *D*_*i *_≥ 0.1**.

In Fig. 3 we show the components of the asymptotic information change *D*_*ik *_for which *D*_*i *_≥ 0.1 holds (149 genes). Blue corresponds to low values (zero) and cyan to high values of *D*_*ik *_(the maximal value of *D*_*ik *_is 20.47). The vertical stripes indicate that the knockout of a few genes effects many other genes whereas most knockouts have only a minor effect on other genes. This is also the reason why we do not show *D*_*ik *_for *N *= 3357 genes because in this case the figure would appear essentially blue.

**Figure 4:**
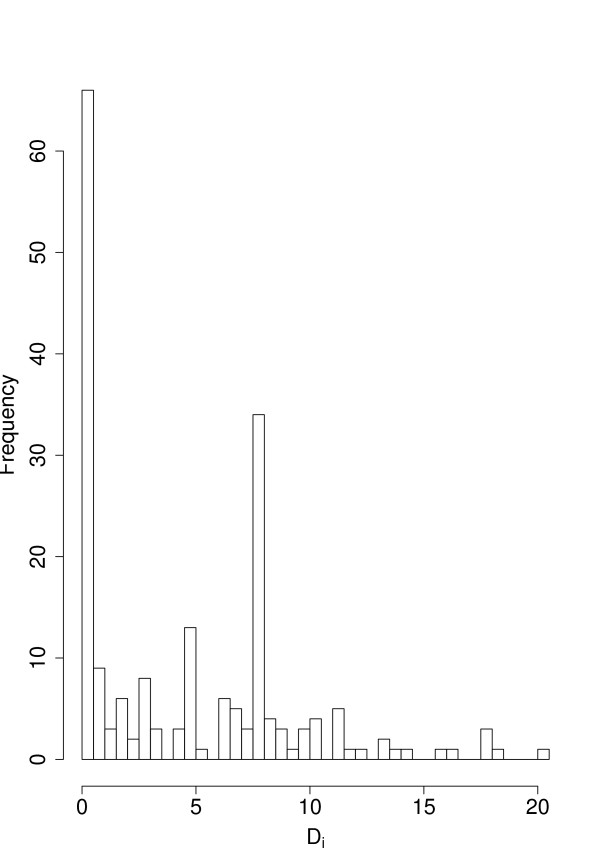
**Histogram of *D*_*i *_for all genes with *D*_*i *_> 0**.

In Fig. 4 we show the histogram of *D*_*i*_. From this figure one can see that the distribution of *D*_*i *_has a heavy tail and that most values are around zero. This indicates that our measure has the desirable property to be very selective by evaluating most perturbations as minor. This corresponds to experimental results showing that only about 10% of all genes in yeast are categorized as *essential *[50] which means that their knockout has a catastrophic influence on the organism.

**Figure 5:**
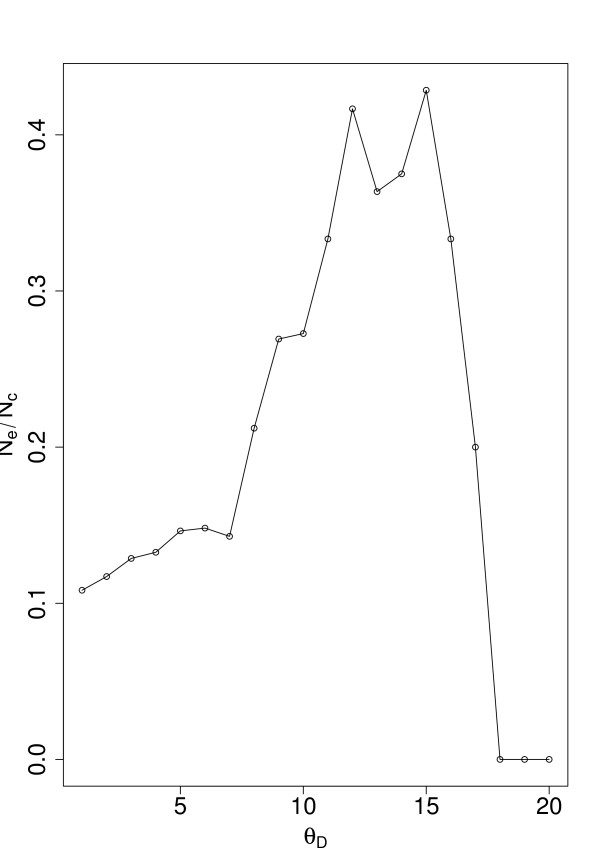
***N*_*e*_/*N*_*c *_in dependence on Θ_*D*_**.

To point out the properties of our measure we show in Fig. 5 results connecting genes with high *D*_*i *_values quantitatively to the appearance of essential genes from gene-deletion experiments in yeast [45]. Figure 5 shows *N*_*e*_/*N*_*c *_in dependence on Θ_*D*_. *N*_*c *_is the number of genes found for which *D*_*i *_> Θ_*D *_holds and *N*_*e *_is the number of essential genes found in this set,

(8) *S*_*c *_= {*i*|*D*_*i *_> Θ_*D*_},

(9) *N*_*c *_= #*S*_*c*_,

(10) *S*_*e *_= {*i*|*i *∈ *S*_*c *_and *i *is essential},

(11) *N*_*e *_= #*S*_*e*_.

**Figure 6:**
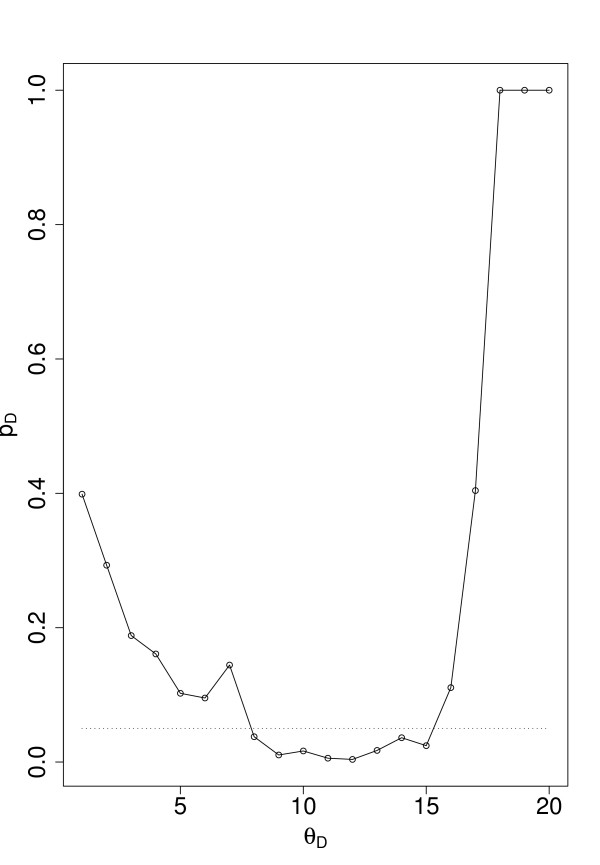
***p_*D *_*in dependence on Θ_*D*_**. The dotted line corresponds to 0.05.

The highest values found by this comparison are over 40%. A natural question arising now is if this occurred just by chance or is this high coverage unlikely to happened accidentally. Figure 6 provides information regarding this question. There we show *p*_*D *_in dependence on Θ_*D*_. The probability *p*_*D *_is the sum of a hypergeometric distribution *p*(*k*; *N, N*_*E*_, *n*) giving the probability to observe exactly *k *essential genes in a set of size *n *= *N*_*c*_(Θ_*D*_) when the total number of genes is *N *containing *N*_*E *_essential genes.

Hence,

(12)

is the probability to observe *k *or more essential genes in the set *N*_*c*_. From Fig. 6 one can see that for Θ_*D *_∈ [8,15] the probability *p*_*D *_< 0.05 (the dotted line corresponds to 0.05). This result suggests that the peaks observed in Fig. 5 do not appear by chance. Further, we obtain possible cut-off values for our gene set to be considered which corresponds to the interval Θ_*D *_∈ [8,15]. From these results we decided to choose  = 12 as cut-off value because for this value *N*_*e*_/*N*_*c *_assumes a maximum value. We call all genes for which *D*_*i *_>  holds *fragile *genes.

In table 1 we show a list of 12 genes found by setting  = 12 for which *p*_*D *_= 0.0039. These genes are ordered according to their out-degree *d*_*out *_in descending order. The first column gives the name of the gene. The second and third, the out- correspondingly in-degree of the gene in the TRN. The forth column gives the value of *D*_*i *_and the fifth column indicates if the gene is found to be essential (Y) or non-essential (N) according to [45]. As one can see, the first gene (YNL216W) is a hub because *d*_*out *_= 240. However, all other genes are not. Interestingly, YNL216W is not an essential gene according to [45]. The results in table 1 demonstrate that our measure does not prefer to select hub genes because only one hub was selected.

**Table 1:** Genes obtained for  = 12.

gene	*d*_*out*_	*d*_*in*_	*D*_*i*_	essential
YNL216W	240	2	17.5	N
YKL043W	92	1	17.9	N
YML007W	89	2	13.0	N
YFR034C	73	2	14.0	N
YER040W	44	1	17.8	Y
YBR112C	26	1	13.6	Y
YPL177C	23	2	13.3	N
YOL148C	13	1	20.4	N
YGL207W	12	2	18.1	N
YLR399C	9	3	12.2	Y
YDR138W	5	1	15.6	Y
YPR072W	4	1	16.1	Y

The first column gives the name of the gene.

The second and third, the out-correspondingly in-degree of the gene in the TRN. The fourth column gives the value of *D*_*i *_and the fifth column indicates if the gene is found to be essential (Y) or non-essential (N) according to [45]. For this set *p*_*D *_= 0.0039.

This underlines the non-trivial characteristics of our measure. For reasons of completeness we show in table 2 the top four knockout genes that cause the largest influence, as measured by

**Table 2:** Top four knockout genes that have the largest impact on other genes.

gene	*d*_*out*_	*d*_*in*_	*D*_*k*_	essential
YML027W	314	2	26.8	N
YGL096W	248	0	97.4	N
YDL056W	129	0	149.8	Y
YHR206W	128	0	27.8	N

First column: gene name. Second column: out-degree. Third column: in-degree. Fourth column: *D*_*k*_. Fifth column: essential genes (yes (Y) or no (N)).

(13)

on other genes. The genes are again ranked according to their out-degrees. All of these genes are hubs. Considering the top 50 genes reveals that in this set 20 genes have an out-degree below 25 and even genes with an out-degree one and two are among these. Again, this demonstrates that hubness is no sufficient property to characterize these genes.

**Figure 7:**
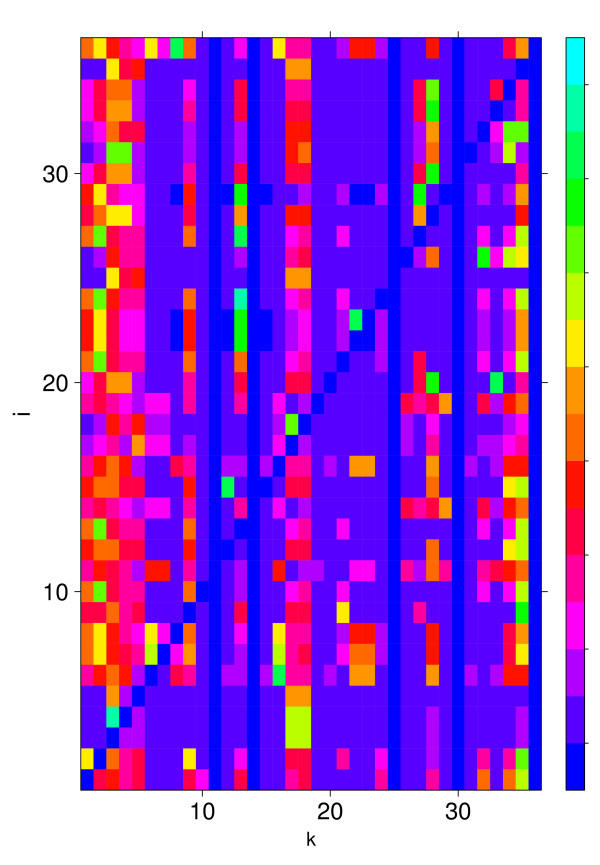
**Asymptotic information change *D*_*ik *_for all genes in the strongly connected component of the transcriptional regulatory network**.

Next, we analyze the structure of the TRN containing our 12 genes shown in table 1. We find that each gene pair is connected (both ways) via a directed path. This implies that the subgraph formed from these 12 genes is part of the strongly connected component of the TRN. As side note we remark that for Θ_*D *_≤ 9 the resulting set of genes is no longer strongly connected and that also for Θ_*D *_= 10 this gene set does not correspond to the entire strongly connected component of the whole TRN comprising *N*_*sc *_= 36 genes. Analysis of the strongly connected component of the whole TRN shows that it contains only 8 essential genes. From this we calculate the probability to find 8 or more essential genes by chance in the strongly connected component. By summing up the probabilities from a hypergeometric distribution we find *p*_*sc *_= 0.021. This shows that essential genes are enriched in the strongly connected component, however, due to *p*_*D *_(Θ_*D *_= 12) <<*p*_*sc *_the strongly connected component represents a less favorable set to identify essential genes than the set found by our method. Fig. 7 shows *D*_*ik *_for the strongly connected component. In contrast to Fig. 3 the influence of the perturbations is now much more severe as can be seen by the many non-blue dots. As a remark we want to remind that the diagonal of *D*_*ik *_is not defined as explained in the methods section.

Finally, we test for the transcriptional regulatory network if our measure and betweenness centrality are similar by calculating Spearman's rank sum correlation coefficient. For the genes in table 1 we obtain a correlation coefficient *r *= 0.0139 and a p-value of 0.965 indicating that the results of both measures are not correlated. Further, we find that among the top 100 ranked genes of both measures only two genes are selected by both measures.

## Discussion

In table 3 we provide some information about the biological processes the genes in table 1 are involved in. All genes found by our measure belong to the category 'regulation of transcription, DNA-dependent'. Further, some additional categories are listed for each gene in table 3. It is apparent that involvement in transcription regulation is the dominating category. This is interesting because the genes listed in the bottom of the table have a fairly low out-degree (see table 1). From our results we hypothesize that the genes found by our method, who have not been declared 'essential' by [45], should be 'fragile' in the sense that they are influenceable quite severely by the malfunctioning of many other genes. Here, 'fragile' should not necessarily be equalized with 'essential' but the organism may be viable, however, it's overall function substantially impaired. It is also important to bear in mind that the list of essential genes used in our analysis is not necessarily complete. After intense literature research we found that YGL207W (also known as SPT16 – subunit of the heterodimeric FACT complex (Spt16p-Pob3p), facilitates RNA Polymerase II transcription elongation) is reported to be essential by [51] confirming our findings.

**Table 3:** Biological processes the genes provided in table 1 participate.

gene	biological process
YNL216W	chromatin silencing at telomere, ribosome biogenesis
YKL043W	pos. regulation of trans. from RNA polymerase II promoter
YML007W	regulation of trans. from RNA polymerase II promoter in response to oxidative stress
YFR034C	response to starvation,
YER040W	positive regulation of transcription
YBR112C	negative regulation of transcription, chromatin remodeling
YPL177C	cellular copper ion homeostasis
YOL148C	chromatin modification, mitotic cell cycle
YGL207W	DNA repair, chromatin modification
YLR399C	DNA repair, chromatin remodeling
YDR138W	response to DNA damage stimulus
YPR072W	protein ubiquitination

In addition to the listed biological processes, all genes are involved in the category 'regulation of transcription, DNA-dependent'.

On a mathematical note we want to remark that the fact that *rank*(*D*_*i*_) and *rank*(*d*_*out*_) respectively *rank*(*d*_*in*_) are correlated, as shown in the beginning of the results section, does not imply that our measure approximates or is even identical to the ranking of the degrees. This can be seen in table 1 because, e.g., the five bottom genes have *d*_*out *_< 20, however, in the whole transcriptional regulatory network are 79 genes that have an out-degree larger than 20. But only seven of them appear in the list.

From a perspective of information processing the connection between asymptotic information change and local network structure represented by their degrees is interesting because it indicates that a local subgraph may be sufficient to study information processing in the overall network. This dissection is interesting because it would allow to reduce the computational complexity considerably that arises studying genomes like yeast or even organisms with more genes. In a former study [27], a similar idea has been proposed in a different methodological framework.

Finally, we want to remark that we repeated the analysis using *D*_*k *_= ∑_*i *_*D*_*ik *_as fragility measure of genes. However, for *D*_*k *_we did not obtain meaningful results regarding the enrichment of essential genes. That means that the information captured by *D*_*ik *_is asymmetric, as one would expect from it's construction.

## Conclusion

In this paper we analyzed the influence that single gene perturbations have on the asymptotic communication abilities of the transcriptional regulatory network of yeast [43,44] to learn about the functional robustness of this network. To study the influence of the perturbations we used an information theoretic measure [36] and approximated the information propagation as a first order Markov chain directly defined for a given network topology. Our numerical studies obtained three major results. First, the asymptotic distributions for the perturbed and unperturbed network states carry implicitly information about their local origin from which the initial signal was transmitted. This confirms results previously found for synthetic networks [36]. Second, using our measure of asymptotic information change we could demonstrate that the predicted set of fragile genes contains a statistically significant enrichment of so called essential genes that are experimentally found to be necessary to ensure vital yeast. Third, a structural analysis of the transcriptional regulatory network revealed that there are significant differences between fragile genes, hub genes and genes with a high betweenness centrality value.

In addition to these findings we consider it to be important to emphasize that we employed graph theoretical, information theoretical as well as statistical methods [52] because the biological information processing in gene networks is unlikely to be treated correctly in a deterministic framework. This demonstrates the power of interdisciplinary approaches and is at the heart of computational systems biology.

## Authors' contributions

All authors contributed to all aspects of the article.
